# High Neutrophil-to-Platelet Ratio Is Associated with Poor Survival in Patients with Acute Aortic Dissection

**DOI:** 10.1155/2022/5402507

**Published:** 2022-06-21

**Authors:** Jungang Pang, Jun Liu, Wantian Liang, Lijun Yang, Liangyin Wu

**Affiliations:** ^1^Department of Cardiology, Yue Bei People's Hospital, Shantou University Medical College, Shaoguan 512025, China; ^2^Medical Research Center and Clinical Laboratory Medicine, Yue Bei People's Hospital, Shantou University Medical College, Shaoguan 512025, China

## Abstract

**Background:**

Acute aortic dissection (AAD), a serious and fatal cardiovascular disease, is characterized by inflammation that may further aggravate the condition. We evaluated the value of the neutrophil-to-platelet ratio (NPR) in the prognosis of AAD.

**Methods:**

We collected records of patients with AAD and clinical data from 2010 to 2020 and followed up on the relevant information for 136 months. The Kaplan–Meier (K–M) survival along with the univariate and multivariate Cox analyses was used to examine the prognostic value of NPR in AAD. In addition, nomograms were constructed by combining NPR, age, Stanford typing, and treatment methods. The accuracy of nomograms was evaluated using calibration plots, and the prediction efficiency of nomograms was evaluated by receiver operating characteristic curve analysis and decision curve analysis (DCA).

**Results:**

The K–M analysis showed that AAD patients with higher NPR exhibited worse prognosis. In addition, different Stanford typing and treatment methods produced varied prognosis results. Univariate and multivariate Cox analyses showed that NPR value, age, classification, and treatment were independent prognostic factors for the overall survival time of patients with AAD. Nomograms constructed by combining NPR, age, Stanford typing, and treatment methods showed good predictive efficacy, and the AUC values for 1-, 3-, and 5-year predicting were 0.82, 0.79, and 0.74, respectively.

**Conclusions:**

Our results suggest that pretreatment NPR can be used as a potential prognostic marker of overall survival time in patients with AAD.

## 1. Introduction

Acute aortic dissection (AAD) is a serious and fatal cardiovascular disease, characterized by intimal tear causing the blood from the cavity to enter the middle of the arterial wall [1]. This finally leads to the separation of the inner layer of the aortic wall [2]. Global epidemiological data show that AAD is more often observed in the elderly over the age of 60 years [3]. The incidence of AAD has increased with the increase in the aging population worldwide [4]. At present, the incidence of AAD is 4.586 for every 100,000 individuals in the age group of 65 to 75 years. The China Registration Study (Sino-RAD) reports that the average age of Chinese patients with ADD is around 51 years, more than 10 years younger than that in European and American countries [5]. Because AAD progresses rapidly, about 24% of patients will die within the first 24 hours and 50% of patients will die within 48 hours if it is not correctly diagnosed and timely intervention measures are not taken in the early stage [6]. According to the Stanford typing, depending on the extent and location of the dissection, AAD can be divided into type A aortic dissection (TAAD) and type B aortic dissection (TBAD), both of which have a high mortality rate [7]. Although certain progress has been made in the diagnosis and treatment of AAD recently due to the improvement in medical technology, the prognosis of the majority of patients with AAD is still not ideal. High-risk factors need to be identified early, and early intervention is important to prevent the progression of the disease and improve the prognosis.

The specific pathogenesis of AAD has remained unclear. Studies have reported that AAD is associated with vascular inflammation and matrix degradation, including chronic infection and the activation of matrix metalloproteinases and the vascular endothelial growth factor [8]. In addition, several studies have reported that markers related to thrombosis and inflammation, including fibrin degradation products, D-dimer, C-reactive protein, and serum amyloid A, are used for the diagnosis and short-term prognosis of AAD [9–12]. Increasing evidence suggests that increased cell count or neutrophil/lymphocyte ratio is an independent adverse prognostic indicator of patients with TAAD [13]. It is known that platelets and neutrophils interact to regulate each other's functions during infection, inflammation, and thrombosis [14]. The recruitment of neutrophils to inflammatory sites is dependent on the mechanism of platelet aggregation. In addition, several studies have reported that the formation of neutrophil–platelet aggregates is associated with thrombosis and vascular inflammation. For instance, Wang et al. reported that the ratio of platelets to neutrophils (PNR) could serve as an independent risk factor for acute ischemic stroke. Moreover, it is associated with early neurological deterioration and a short-term prognosis following intravenous thrombolysis [15]. However, the value of NPR in the long-term prognosis of AAD has not yet been reported.

In the present study, we retrospectively collected the clinical data of patients with AAD in the recent 10 years and conducted a detailed follow-up to explore the effects of preoperative NPR on the overall survival of patients with AAD.

## 2. Materials and Methods

### 2.1. Patient Collection

This retrospective study collected the record of patients treated in Yue Bei People's Hospital from January 1, 2010, to December 31, 2020, and followed up on their relevant data until May 2021 to collect the relevant clinical information. The diagnosis of AAD is primarily based on the treatment and diagnosis guidelines for aortic diseases proposed by the European Society of Cardiology (ESC) 2014, whose classification standard is based on the Stanford standard as per the anatomical classification. The exclusion criteria were as follows: the time of onset was more than 14 days or was unknown, incomplete information, recurrent AAD, hematological diseases, cirrhosis, systemic lupus erythematosus (SLE), Marfan syndrome, traumatic AAD, malignant tumor, AIDS, and incomplete data. Finally, 309 patients were included in the study. The research scheme was approved by the Ethics Committee of Yue Bei People's Hospital (KY-2020-166). Because of the retrospective nature of the study, informed consent was not required.

### 2.2. Data Collection

The first blood cell test results of patients with AAD were collected. Stanford typing was used to classify the patients, and the treatment methods received were recorded. NPR is defined as the absolute value of neutrophil count divided by the absolute value of platelet count. To facilitate scientific counting, NPR = number of neutrophils/number of platelets × 10.

### 2.3. Statistical Analysis

All statistical analyses were performed using the R software (version 4.1.1). Kaplan–Meier survival analysis was performed using the “survival” and “surviviner” packages, and the “survival” package was used to perform univariate and multivariate Cox analyses. A *P* value < 0.05 was considered significant. The “RMS” package was used to build nomograms, “survivalroc” was used for the receiver operating curve (ROC) analysis, and the “decisioncurve” package was used for decision curve analysis (DCA).

## 3. Results

### 3.1. Baseline Characteristics of Patients

A total of 309 patients with AAD were included in this study, of which 121 died during follow-up (39.2%). Statistics showed that 262 men accounted for 84.8% of the total patients. Among all cases, 204 cases were diagnosed as TBAD, accounting for 66% of the total. Among these patients, 123 cases were treated with thoracic endovascular aortic repair (TEAR), accounting for 39.8% of the patients. In addition, 47 patients used the traditional surgical treatment (ST), accounting for 15.2% of cases, and 139 patients were treated with conservative medication (CM), accounting for 45% of the total. The specific clinical information of the study population is summarized in Table 1.

### 3.2. NPR Was Associated with Overall Survival

**Figure 1:**
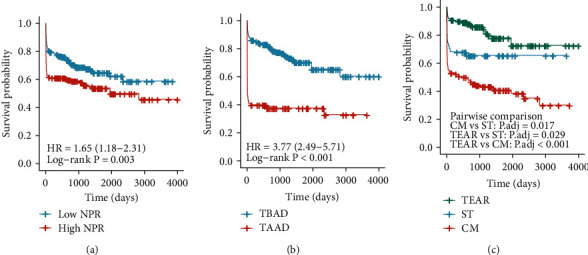
Kaplan–Meier survival curve analysis. (a) Patients with acute aortic dissection were stratified according to the NPR value, and the difference in the overall survival time was calculated using the Kaplan–Meier analysis. The Kaplan–Meier curve shows the difference in the overall survival time between (b) type A aortic dissection (TAAD) and type B aortic dissection (TBAD) and (c) treatment methods. The p.adj is obtained by multiple hypothesis test using Bonferroni method to correct the significance level. TEAR: thoracic endovascular aortic repair; CM: conservative management; ST: surgical treatment.

The median value of NPR showed that the study population was divided into high- and low-risk groups, and the difference in the overall survival between the two groups was calculated using the K–M analysis. The results showed that patients with AAD in the high-NPR group had worse overall survival time than those in the low-NPR group (log-rank test, *P* = 0.003; Figure 1(a)). Simultaneously, the difference in the overall survival time between two different subtypes of TAAD and TBAD was analyzed. Patients with TAAD had a shorter overall survival expectation compared with TBAD (log-rank test, *P* < 0.001; Figure 1(b)). In addition, the effects of different treatment methods on the overall survival time of patients with AAD were analyzed. The results showed that patients treated with thoracic endovascular aortic repair had the best prognosis as compared with other patients. Compared with surgical treatment and conservative drug treatment, the latter had relatively the worst prognosis, with significant differences among the three treatment methods (Figure 1(c)).

### 3.3. NPR Is an Independent Prognostic Indicator

**Figure 2:**
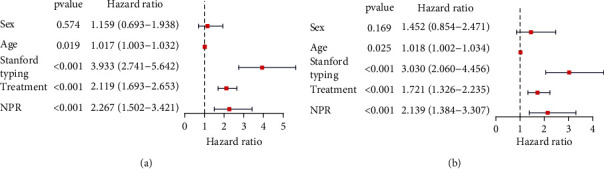
Prognostic correlation analysis. (a) Univariate and (b) multivariate Cox regression analyses identified NPR as an independent prognostic factor for acute aortic dissection.

**Figure 3:**
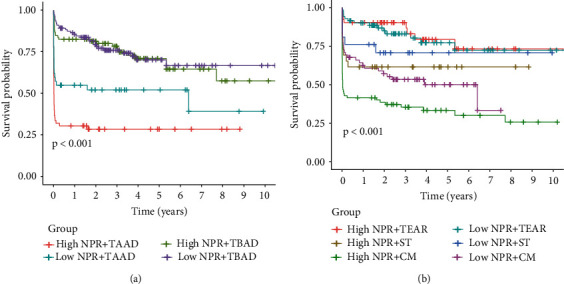
Two-factor Kaplan–Meier survival analysis. (a) Overall survival analysis combined with NPR and typing. (b) Overall survival analysis combined with NPR and treatment. TAAD: type A aortic dissection; TBAD: type B aortic dissection; TEAR: thoracic endovascular aortic repair; CM: conservative management; ST: surgical treatment.

To further explore the prognostic factors related to the overall survival time of AAD, a univariate Cox regression analysis was performed on variables including sex, age, treatment mode, Stanford typing, and NPR. The results showed that age, treatment, Stanford typing, and NPR were associated with the overall survival time of patients with AAD (Figure 2(a)). Subsequently, multivariate Cox analysis revealed that age, treatment mode, Stanford typing, and NPR were independent prognostic factors for patients with AAD (Figure 2(b)). To further clarify the relationship between NPR and Stanford typing, we performed a two-factor K–M survival analysis. The results showed that patients with both TAAD and a high NPR had the worst overall survival time, and those with TBAD and low NPR had the best prognosis (Figure 3(a)). Next, the relationship between NPR and different treatment methods was analyzed. The K–M survival analysis revealed that patients with a high NPR and treated with conservative management (CM) presented the worst prognosis, whereas those treated with thoracic endovascular aortic repair (TEAR) had a relatively good prognosis (Figure 3(b)).

### 3.4. Nomogram Construction

**Figure 4:**
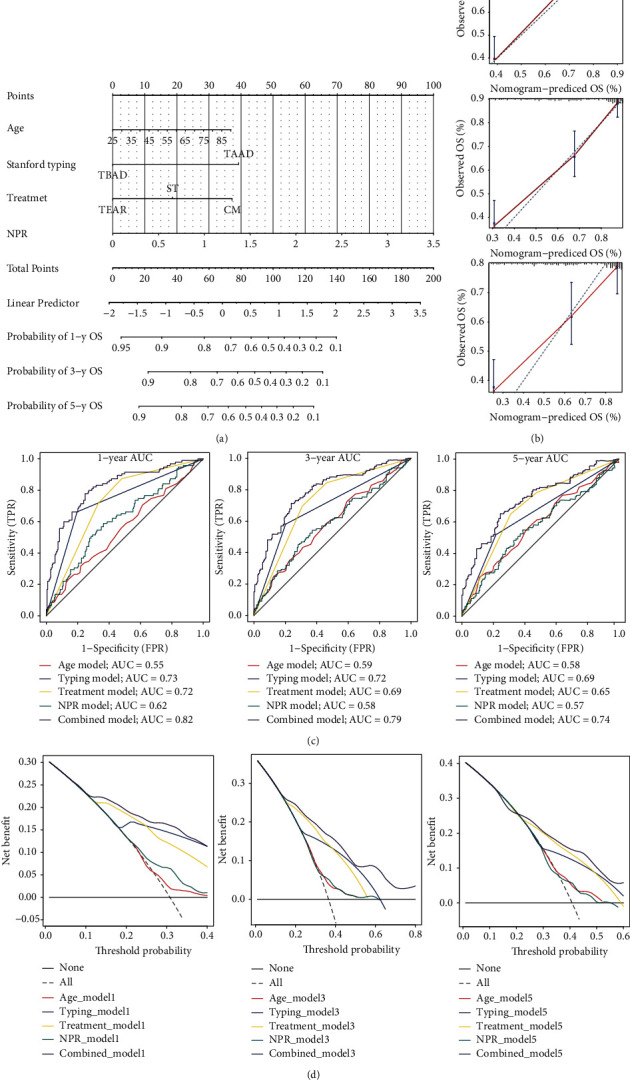
Nomograms of risk factors related to the prognosis of acute aortic dissection were constructed. (a) Nomogram for predicting 1-, 3-, and 5-year overall survival in patients with acute aortic dissection (AAD). (b) Calibration curve for predicting 1-, 3-, and 5-year survival time in patients with acute aortic dissection. (c) The receiver operating characteristic curve (ROC) of 1-, 3-, and 5-year overall survival was calculated. (d) The decision curve analysis (DCA) curves of different models for 1-, 3-, and 5-year overall survival prediction were shown. TEAR: thoracic endovascular aortic repair; CM: conservative management; ST: surgical treatment.

We established a convenient method for clinical application to predict the survival probability of patients with AAD. A nomogram was constructed to predict the 1-, 3-, and 5-year overall survival of patients with AAD. The nomogram included independent prognostic factors for AAD, including age, mode of treatment, Stanford typing, and NPR (Figure 4(a)). Next, the accuracy and discrimination of the nomogram were evaluated using calibration plots, in which the 45-degree dotted line represented the ideal discrimination. The red solid line represented the actual prediction performance of the nomogram. Calibration plots revealed that the prediction ability of the nomogram was extremely close to the ideal discrimination, showing good prediction performance (Figure 4(b)). The predictive efficacy of the nomogram on the overall survival time of AAD was evaluated using the ROC curve. The area under the curve (AUC) of 1-, 3-, and 5-year survival was 0.82, 0.79, and 0.74, respectively (Figure 4(c)). The clinical usefulness of the nomogram was further evaluated by DCA, showing the best net benefit (Figure 4(d)). These results suggested the nomogram to be a better predictor of the overall survival of patients with AAD than either marker alone.

## 4. Discussion

Acute aortic dissection is a highly severe cardiovascular disease associated with high mortality. Studies have reported that up to 21% of patients with acute type A aortic dissection died before reaching the hospital [16]. Although certain progress has been made in the diagnosis and treatment of AAD due to the improvement in medical technology recently, the prognosis of the majority of patients with AAD is still not ideal with low overall survival time. AAD is characterized by acute onset, atypical clinical manifestations, and rapid progression. However, the pathogenesis and prognostic factors need to be clarified. Thus, determining the related overall survival index of AAD is clinically significant to improve its prognosis.

We found that the results of the first blood cell examining following admission had important prognostic value for patients with AAD. The K–M survival analysis revealed that NPR was associated with the overall survival of patients with AAD. Those with a higher NPR had a worse prognosis. In addition, we compared the differences in the overall survival time between different treatment methods and Stanford typing. The K–M survival analysis showed that patients with TAAD had a worse prognosis than those with TBAD, which was consistent with the previously reported results [17, 18]. Patients treated with thoracic endovascular aortic repair had the best prognostic effect, and those treated with conservative drugs showed the worst prognostic effect. Interestingly, the results showed that different surgical treatments impacted the overall survival of patients. We further verified the value of NPR in the prognosis of AAD using a combination of univariate and multivariate Cox regression analyses. The results showed that it was independent of sex, age, treatment, and Stanford typing and could serve as an independent prognostic factor of AAD. We proposed NPR as a new index in AAD that suggested thrombosis and inflammatory response to a certain extent. In addition, it reflected the balance between neutrophils and platelets. At present, NPR value has not been reported in the prognosis of AAD. The clinical correlation analysis of 400 patients with acute ischemic stroke by Jin et al. stated that platelet-to-neutrophil ratio (PNR) was related to the 3-month prognosis of acute ischemic cerebral infarction and was an independent prognostic protective factor [19]. The results of Hong et al. showed that both PNR at admission and PNR 24 hours following intravenous thrombolysis of ischemic stroke were associated with poor prognosis at 3 months. The ROC analysis showed its reliability as a prognostic index [20]. Our results suggested that NPR could be used as a novel adverse prognostic indicator in patients with AAD.

The occurrence of AAD is related to inflammation, which stimulates the necrosis and apoptosis of smooth muscle cells, causing the degradation of elastic tissue and aortic dissection. C-reactive protein (CRP) and serum amyloid A (SSA)—acute phase reactants in the serum—have been used in the early diagnosis and prognosis of several diseases [21]. Wen et al. reported CRP as an important risk factor for AAD and was associated with independent death during hospitalization in patients with AAD [22]. In addition, Yuchen et al. reported significantly high levels of SSA in patients with AAD than in healthy controls. Thus, SSA was an independent prognostic factor in patients with AAD during hospitalization [23]. This study suggested that patients with elevated NPR had a worse long-term prognosis; the two-factor survival analysis showed that a high NPR had a certain prognostic value in different types and treatment methods. A high NPR implied a relatively higher neutrophil value and higher inflammatory reaction, which is also consistent with the progression of AAD promoted by inflammatory reactions. Studies have shown that AAD stimulates the release of chemokines in the adventitia of dissecting aorta to mobilize neutrophils to recruit to peripheral blood [24]. In addition, excessive neutrophil infiltration increases the release of metalloproteinase 9, consequently promoting the deterioration of the interlayer [25]. Platelets are known to activate the coagulation system and thrombosis. The occurrence of AAD has been related to the activation of the coagulation system. D-dimer, fibrinogen, and fibrin products show important diagnostic and prognostic values in patients with AAD [12, 26, 27]. AAD results in the formation of hematomas, causing platelet activation and adhesion to the torn vessel wall. Activated platelets release platelet particles to recruit the inflammatory cells and interact with the activated platelets, further aggravating vascular inflammation and causing tissue damage [28]. The interaction between neutrophils and platelets, on the one hand, promotes the inflammatory reaction during thrombosis; on the other hand, it results in the consumption of platelets. Neutrophils are recruited from the bone marrow to the peripheral blood, leading to a change in NPR. Overall, these results suggest that NPR is clinically significant in the development of AAD. Combined NPR with Stanford typing analysis indicated that AAD patients with high NPR and TAAD presented the worse overall survival than patient with low NPR and TAAD. This may signal that it is important to pay attention to the changes of thrombus inflammation in the treatment of TAAD patients. Multiple studies have highlighted the critical role of inflammation pathways and platelet activation in the progression of AAD [29–31]. This suggests that inhibition of inflammation and platelet activation may be a novel therapeutic strategy to improve the long-time survival of AAD patients in the future.

The levels of neutrophils and platelets are routine items in the clinic and can be easily obtained. To further facilitate the application of NPR in clinical evaluation, a nomogram was constructed by combining age, classification, and treatment methods, which are independently related to the overall survival time of AAD. ROC and DCA analyses confirmed the reliability of the nomogram as a prognostic index.

This study still had certain limitations. First, this study was based on single-center clinical research. Further multicenter research is conducive to enhancing the credibility of the results. Second, this study was based on a retrospective study; a prospective cohort study in the recent 10 years will be more helpful to verify the prognostic value of NPR in AAD.

## 5. Conclusion

NPR is a routine and easily accessible inflammatory biomarker in the clinical detection of AAD. This study showed that a high NPR value can serve as an independent adverse risk factor for the long-term prognosis of AAD. Nomogram constructed in combination with NPR, age, classification, and treatment is conducive to the hierarchical management of patients.

## Figures and Tables

**Table 1:** Baseline characteristics and clinical data of patients with AAD.

Characteristic	Levels	Overall
*n*		309
Status, *n* (%)	Alive	188 (60.8%)
	Dead	121 (39.2%)
Sex, *n* (%)	Female	47 (15.2%)
	Male	262 (84.8%)
Stanford, *n* (%)	B	204 (66%)
	A	105 (34%)
Therapy, *n* (%)	PCI	123 (39.8%)
	ST	47 (15.2%)
	CM	139 (45%)
Age, median (IQR)		55 (48, 64)
WBC, median (IQR)		12.48 (9.11, 15.61)
PLT, median (IQR)		181 (148, 219)
GRAN, median (IQR)		10.07 (6.87, 13.16)
LYM, median (IQR)		1.28 (0.89, 1.8)
RBC, median (IQR)		4.46 (3.99, 4.88)
HGB, median (IQR)		132 (117, 143)

WBC: white blood cell; PLT: platelet; GRAN: neutrophilic granulocyte; LYM: lymphocyte; RBC: red blood cell; HGB: hemoglobin.

## Data Availability

Data can be made available by contacting the corresponding author.
